# The Effects of Acute Dopamine Precursor Depletion on the Reinforcing Value of Exercise in Anorexia Nervosa

**DOI:** 10.1371/journal.pone.0145894

**Published:** 2016-01-25

**Authors:** Caitlin B. O’Hara, Alexandra Keyes, Bethany Renwick, Marco Leyton, Iain C. Campbell, Ulrike Schmidt

**Affiliations:** 1 King’s College London, Institute of Psychiatry, Psychology & Neuroscience, Department of Psychological Medicine, Section of Eating Disorders, London, United Kingdom; 2 Department of Psychiatry, McGill University, Montreal, QC, Canada; Charité-Universitätsmedizin Berlin, Campus Benjamin Franklin, GERMANY

## Abstract

This study investigated whether dopaminergic systems are involved in the motivation to engage in behaviours associated with anorexia nervosa (AN), specifically, the drive to exercise. Women recovered from AN (AN REC, n = 17) and healthy controls (HC, n = 15) were recruited. The acute phenylalanine/tyrosine depletion (APTD) method was used to transiently decrease dopamine synthesis and transmission. The effect of dopamine precursor depletion on drive to exercise was measured using a progressive ratio (PR) exercise breakpoint task. Both groups worked for the opportunity to exercise, and, at baseline, PR breakpoint scores were higher in AN REC than HC. Compared to values on the experimental control session, APTD did not decrease PR breakpoint scores in AN REC, but significantly decreased scores in HC. These data show that women recovered from AN are more motivated to exercise than HC, although in both groups, activity is more reinforcing than inactivity. Importantly, decreasing dopamine does not reduce the motivation to exercise in people recovered from AN, but in contrast, does so in HC. It is proposed that in AN, drive to exercise develops into a behaviour that is largely independent of dopamine mediated reward processes and becomes dependent on cortico-striatal neurocircuitry that regulates automated, habit- or compulsive-like behaviours. These data strengthen the case for the involvement of reward, learning, habit, and dopaminergic systems in the aetiology of AN.

## Introduction

Anorexia nervosa (AN) is a serious disorder associated with high rates of death and disability. Core symptoms include a relentless pursuit of thinness via extreme food restriction and other driven behaviours, such as excessive exercise. Treatment is difficult, due, in part, to the ego-syntonic or rewarding nature of symptoms [1]. Experiencing reward involves interactions between hedonic ‘liking’, motivational ‘wanting’, and learning. ‘Wanting’ involves incentive salience attribution which leads reward-related stimuli to become motivationally attractive: this primarily involves mesolimbic dopaminergic systems. These also mediate locomotor activity, behavioural activation, and motivational drives including the motivational drive to eat [2]. Alterations in these circuits are implicated in the development/maintenance of core AN symptomatology [3, 4].

Individuals with AN show reduced cerebrospinal fluid homovanillic acid, raising the possibility of a trait-related disturbance in DA metabolism in AN [5]. This is partially supported by studies reporting associations between dopaminergic genes and vulnerability to illness [6, 7]. People recovered from AN are reported to have increased dopaminergic D2/D3 receptor binding potential in the anterior-ventral striatum, suggesting that decreased DA levels or increased D2/D3 receptor density or affinity is associated with AN [8]. Neuroimaging has indicated that people recovered from AN lack differential activation in reward-related neurocircuitry in response to monetary wins and losses [9]. Moreover, fMRI investigations indicate that individuals with AN show increased responses to AN-related cues (underweight body [10] and taste-reward [11, 12] stimuli) in the ventral striatum, a DA-innervated brain region implicated in reward. These studies associating DA function with AN are supported by animal studies which show that chronic food restriction sensitises the mesolimbic DA system [13].

Data on aberrant reward processing in AN suggest that heightened sensitivity in dopaminergic circuits is related to its pathophysiology [3]. Importantly, this is currently a conceptual model of AN; in fact, there are studies showing conflicting results or no abnormality in DA function (e.g., [8, 14, 15]) and further research is needed. Nevertheless, a recent model hypothesises that increased DA sensitivity to illness-compatible stimuli (e.g., exercise) heightens their incentive salience, promoting AN-associated behaviours which over time become compulsive and habitual [4]. Reward-associated dopaminergic systems may then become less important in maintaining illness. However, this model is relatively simple as DA-associated reward systems are complex. For example, data implicate prefrontal cortical and ventral striatal circuits in regulating goal-directed actions, whereas automated, habit-like behaviours are under more dorsal-striatal control and eventually, may become DA-independent [16–19]. The development of both behaviours is therefore influenced by dopaminergic processes, but expression of the latter may be less susceptible to change when DA is altered.

A heightened drive to exercise is common in AN and the rewarding nature of exercise has been highlighted since the earliest descriptions of illness [20]. Exercise drive is higher in sufferers relative to both anxious and healthy controls (HC) [21], and is associated with poorer outcome, increased relapse, and extended hospitalisation [22–24]. However, the neurobiology of exercise-related reward in AN remains under researched. Rodent models of AN [activity-based anorexia (ABA)] are based on the observation that some rats will work for the opportunity to run [25]. Rats that are susceptible to ABA express increased DA D2 receptor levels in the caudate-putamen, suggesting decreased dopaminergic functioning or increased receptor density or affinity [26]. DA antagonists, in comparison, reduce activity and weight loss, and increase food intake in ABA [27]. Individual differences in the effects of lowered DA can also be seen, and depleting DA stores reduces preference for running in animals with low but not high experience of exercise [28].

The processes described above are also implicated in the desire to exercise in humans. Giel et al. [29] reported that patients with AN and athletes attribute more attention and subjective pleasure towards exercise-related stimuli than non-athletes. They also found that exercise-related stimuli induced greater activation of the prefrontal cortex in individuals with AN relative to athletes and non-athletes, suggesting more inhibitory control is required during exposure to exercise cues [30]. Finally, the reinforcing value of exercise can be quantified using a progressive ratio (PR) task. The PR and related tasks are well-established tools developed by the field of behavioural economics, which assess motivation to obtain a range of rewards in terms of the amount of “work” an individual is willing to expend to earn access to it [31, 32]. In the context of physical activity, the PR task measures the reinforcing value of exercise by requiring participants to expend progressively increasing amounts of effort (*i*.*e*., computer key presses) for successive minutes of exercise. While perhaps not an obvious measure of the motivation to do physical exercise in humans, it has been demonstrated that, using this paradigm, individuals with AN will expend considerable effort for the opportunity to exercise; however, absence of a control group precluded determining whether motivational drive to exercise was altered [33]. Together, the above findings suggest that activity-related stimuli can acquire incentive value in AN.

Acute phenylalanine/tyrosine depletion (APTD) is used to assess the relationship between reduced DA function and behaviour [34]. In humans, APTD is associated with reduced reward sensitivity [35] and diminished ability to sustain motivation to obtain reward using a PR breakpoint task [31, 32, 36]. It also impairs frontostriatal functional connectivity during set-shifting [37], a measure of cognitive rigidity (*i*.*e*., a cognitive style implicated in compulsivity [17]), and preliminary data suggest that it may favour reliance on habitual control at the expense of competing goal-directed actions [38]. However, these latter findings are inferred from computational learning methods; thus, statements made about habitual behaviour in a natural environment based on these tasks should be interpreted with caution.

We used APTD to investigate whether lowering DA influences motivation to work for exercise using a PR exercise breakpoint task in women recovered from AN (AN REC) and HC. Individuals with acute AN have widespread alterations in central- and peripheral-organ systems. This is a major research confound in that it becomes difficult to determine whether changes are a cause or consequence of starvation. Importantly, however, women recovered from AN often show incomplete normalisation of illness-related behaviours as well as altered neural reward processing of food cues [9, 39–41], suggesting that these alterations may reflect premorbid traits that contribute to illness vulnerability. Thus, to avoid confounding effects of malnutrition and to investigate state- vs trait-aspects of illness, we recruited AN REC and HC. Given suggestions that there is reduced DA transmission in the regulation of habitual vs motivated behaviour (e.g., [19, 38, 42]) together with data linking compulsive exercise to illness course and vulnerability to AN [20], we hypothesised that APTD would not affect willingness to work for exercise in individuals recovered from AN, but would in HC.

## Materials and Methods

### Participants

Nineteen adult women recovered from AN (AN REC) were recruited via Beat, the UK’s eating disorder (ED) charity, and using a King’s College London circular e-mail. Recovery was defined as: (i) maintaining weight >85% of average body weight, (ii) not having binged, purged, or engaged in significant restrictive eating patterns and/or other compensatory behaviours, all for at least 1 year before the study, and (iii) no clinically significant scores (≥2.80) on the Eating Disorders Examination Questionnaire (EDE-Q) [43]. Seventeen HC women were recruited using a University circular e-mail. Controls were matched for age and body mass index (BMI), and reported no history of an ED or any other psychiatric illness. Of the 36 women recruited, four did not complete the study: three (2 AN REC, 1 HC) withdrew due to inability to ingest, or sickness, following the amino acid (AA) drink, and the fourth (HC) relocated. Seventeen AN REC and 15 HC were included in the analysis.

Exclusion criteria were: insufficient knowledge of English, a significant medical illness (e.g., a cardiovascular or neurological disorder), substance abuse/dependence (including smoking >10 cigarettes/day), presence of an Axis I psychiatric disorder needing treatment in its own right, and pregnancy. AN REC participants who were taking selective serotonin reuptake inhibitors (SSRIs) were not excluded, provided they had been on a stable dose for at least 3 months. This study was approved by the London/West London Research Ethics Committee (ref 11/LO/1082). Informed written consent was obtained from all volunteers.

### Procedure

Two sessions were scheduled a minimum of three days apart. On the day prior to testing, participants followed a low protein diet and were asked to fast and abstain from caffeine and/or smoking from midnight. Consumption of alcohol was forbidden in the 24h preceding testing. On the morning of each session, height and weight were measured, and baseline questionnaires assessing eating behaviour, exercise behaviour, mood, and reward/punishment sensitivity were completed. Blood (5ml) was used for analysis of baseline plasma AA.

Participants then ingested one of two AA mixtures, one deficient in DA’s precursors, phenylalanine and tyrosine (APTD), or one nutritionally balanced control mixture (BAL). Composition and administration of the AA mixtures were based on previous methods [34]. Participants were allocated at random to either BAL (AN REC: 47.1%, HC: 46.7%) or APTD (AN REC: 52.9%, HC: 53.3%) first, in accordance with a counterbalanced cross-over design to control for order effects. Volunteers and researchers were blind to treatment allocation. Manufacturing and randomisation of the drinks was conducted by the Royal Victoria Infirmary pharmacy (Newcastle-Upon-Tyne).

Four hours after ingestion of the AA mixture, blood was drawn to measure plasma concentrations of AAs. This was followed by the PR exercise breakpoint task. Tyrosine and phenylalanine plasma levels were measured as an index of the extent of APTD using high-performance liquid chromatography and fluorometric detection (HyPURITY, Thermo Electron Corporation). Samples were missing from 3 HC and 4 AN REC participants. Participants were compensated with £100 for time and effort.

### Self-Report Questionnaires

Baseline self-report ratings included the EDE-Q [43], the Temperament and Character Inventory Revised (TCI-R) [44], and the Behavioral Inhibition/Behavioral Activation Scales (BIS/BAS) [45] to measure trait conceptualisations of reward and punishment sensitivity. Baseline exercise behaviour was assessed using self-reported weekly exercise (hours/week) and the Reasons for Exercise Inventory (REI) [46], a 24-item self-report scale assessing motivations to exercise for reasons of weight control, improving physical attractiveness, improving body tone, fitness, health, improving mood, and enjoyment.

Self-report questionnaires assessing changes in mood pre- and post-exercise were: the Depression, Anxiety, and Stress Scales (DASS-21) [47] and the Profile of Mood States-Bipolar Form (POMS-BI) [48]. Unipolar VAS were used to assess urge to exercise, nausea, and hunger immediately before the PR exercise task. VAS items were rated using 10cm lines (e.g., 0: “Not Hungry” and 10: “Extremely Hungry”).

### Progressive Ratio (PR) Exercise Breakpoint Task

Four hours after AA ingestion, participants were offered the opportunity to work for up to 30mins of exercise [using an interactive exercise game (Xbox 360, Your Shape: Fitness Evolved 2012)] on a computerised PR schedule. Participants were instructed that the number of computer key presses to produce reinforcer delivery increased systematically within a session until their response behaviour ceased or did not meet the criterion level (the breakpoint). Instructions described that each session consisted of 10 trials, with 1/10^th^ of the maximum amount of game time (3mins) earned at each. To earn a reinforcer, participants pressed the keys ‘a’ and ‘w’ for an unknown predetermined number of times. The first 3 minutes of exercise required 25 key presses, and each subsequent reinforcer required a response number equal to 1.8 times the previous ratio (i.e., 45, 81, 146, 262, 472, 850, 1 531, 2 755 and 4 959 presses for the remaining nine ratios). Once a ratio was completed, participants engaged in 3mins of exercise. Individuals were then given the option to continue working for additional reinforcers. Participants were told that exercise trials could be earned, played, or stopped at any time during the session, and that there was no requirement to engage in the task. They were aware that if they chose not to work for all 30mins of exercise they would be required to stay in the testing room for the remainder of the time. Procedures were based on studies in addiction and AN [32, 33].

### Sample Size Calculation

An *a-priori* power calculation with PR exercise breakpoint score (number of key presses given for the last reinforcer received) as the primary outcome measure, and based on a repeated measures ANOVA design, determined that a total sample size of 26 would have 95% power to detect a medium effect size of 0.30 with a 0.05 two-sided significance level. Adding a drop-out correction factor (1/1-a) with attrition a = 0.10 per group, we aimed to recruit a sample size of 16 participants/group (17 AN REC and 15 HC completed the study).

### Statistical Analyses

Statistical analyses used SPSS Statistics for Windows. An alpha level of 0.05 was used for all tests, which were two-tailed. Logarithmic transformations and/or robust bootstrapping equation methods based on 1000 bootstrap samples were used when assumptions of normality and homogeneity of variances were violated.

Independent samples t-tests were used to compare group differences in clinical characteristics and self-report questionnaires. A repeated measures ANOVA with Drink (BAL, APTD) as the within-subjects factor, and Group (AN REC, HC) as the between-subjects factor, was conducted to investigate within- and between-group differences in PR exercise breakpoint scores in the two AA conditions. To control for possible relationships between willingness to work for exercise and potential confounds, and to better understand the relationship between drive for exercise and sensitivity to APTD, the difference (for an individual) between PR exercise breakpoint scores during the BAL and APTD conditions was correlated with eating pathology, baseline exercise experience, mood, achievement orientation (e.g., drive and persistence), urge to exercise, hunger, and nausea. *Post-hoc* t-tests were corrected for multiple comparisons using Bonferroni corrections. Means ± SD are reported; Cohen’s d and partial eta squared (*η^2^*) effect sizes (ES) are given for independent samples t-tests and ANOVAs, respectively.

## Results

### Baseline Characteristics

No significant group differences with respect to age, ethnicity, education, BMI, or exercise (hours/week) were observed. Relative to HC, AN REC had higher eating pathology and mood pathology (EDE-Q and DASS-21 scores); however, none scored within the clinical range. AN REC also rated “improving tone” as a more important reason to exercise compared to HC (Table 1), and showed increased harm avoidance (TCI-R scores) and increased sensitivity to punishment (BIS scores) (S1 Table). *A priori* exploratory analysis showed no group differences in exercise breakpoint scores between AN REC who were taking antidepressants (n = 9) and those who were not (n = 8) [F(1, 15) = 2.42, p = 0.15, *η^2^* = 0.14] and thus analyses involve all AN REC.

**Table 1:** Participant Baseline Characteristics.

Baseline Characteristics	AN REC (n = 17)	HC (n = 15)	Statistics: AN REC to HC
Age, years	24.65 ± 5.24	23.14 ± 3.18	t (30) = -0.97, p = 0.34, ES = 0.35
Ethnicity	White: 11 Black: 0 Hispanic: 1 Other: 2	White: 11 Black: 1 Hispanic: 1 Other: 2	χ^2^(3) = 1.25, p = 0.75
Education, years	17.82 ± 2.92	17.20 ± 2.08	t (30) = -0.69, p = 0.50, ES = 0.25
BMI, kg/m^2^	21.45 ± 2.13	21.74 ± 1.58	t (30) = 0.57, p = 0.67, ES = 0.21
Exercise, hours/week	3.69 ± 2.52	2.64 ± 1.46	t (28) = -1.36, p = 0.18, ES = 0.50
EDE-Q, Restraint**	1.50 ± 1.09	0.35 ± 0.39	t (30) = -4.07, p < 0.01, ES = 1.49
EDE-Q, Eating Concern**	0.75 ± 0.53	0.09 ± 0.18	t (30) = -4.80, p < 0.01, ES = 1.76
EDE-Q, Weight Concern**	1.73 ± 1.39	0.43 ± 0.38	t (30) = -3.71, p < 0.01, ES = 1.21
EDE-Q, Shape Concern**	2.24 ± 1.62	0.67 ± 0.61	t (30) = -3.71, p < 0.01, ES = 1.21
EDE-Q, Global Score**	1.55 ± 1.01	0.38 ± 0.34	t (30) = -4.45, p < 0.01, ES = 1.63
DASS-21, Depression*	5.94 ± 6.12	2.13 ± 3.38	t (30) = -2.14, p = 0.04, ES = 0.78
DASS-21, Anxiety**	4.94 ± 5.01	0.93 ± 1.67	t (30) = -3.11, p < 0.01, ES = 1.14
DASS-21, Stress**	15.76 ± 8.91	6.80 ± 6.35	t (30) = -3.24, p < 0.01, ES = 1.19
DASS-21, Total Score**	26.65 ± 16.43	9.87 ± 10.18	t (30) = -3.42, p < 0.01, ES = 1.25
REI, Weight Control	4.59 ± 1.50	3.63 ± 1.49	t (30) = -1.82, p = 0.08, ES = 0.67
REI, Attractiveness	3.57 ± 1.42	3.88 ± 1.42	t (30) = 0.61, p = 0.55, ES = 0.22
REI, Tone**	4.55 ± 1.58	2.73 ± 1.57	t (30) = -3.25, p < 0.01, ES = 1.19
REI, Health	4.57 ± 1.18	5.16 ± 1.50	t (30) = 1.22, p = 0.23, ES = 0.45
REI, Fitness	5.13 ± 1.13	4.93 ± 1.18	t (30) = -0.49, p = 0.63, ES = 0.18
REI, Mood	4.60 ± 1.21	4.00 ± 1.65	t (30) = -1.17, p = 0.25, ES = 0.43
REI, Enjoyment	2.49 ± 1.04	2.76 ± 1.80	t (30) = 0.53, p = 0.60, ES = 0.19

*Legend*: AN REC: anorexia nervosa recovered. BMI: body mass index. DASS: Depression, Anxiety, and Stress Scales. EDE-Q: Eating Disorders Examination Questionnaire. ES: Cohen’s d effect size. HC: healthy controls. REI: Reasons for Exercise Inventory. SD: standard deviation. Data are expressed as Means ± SD.

** *P ≤ 0*.*01*.

* *P ≤ 0*.*05*.

Significant Drink by Time interactions indicated that plasma tyrosine and phenylalanine decreased significantly 4h post-ingestion of the APTD AA mixture (Table 2). It decreased phenylalanine and tyrosine levels by 80.93% and 73.60%, respectively, in HC, and to 78.43% and 73.14%, in AN REC. In contrast, the BAL mixture increased phenylalanine and tyrosine levels by 242.15% and 248.15% in HC, and to 293.33% and 257.30%, in AN REC.

**Table 2:** Plasma Phenylalanine (PHE) and Tyrosine (TYR) Concentrations (μmol/l).

Plasma Amino Acids	AN REC (n = 13)	HC (n = 12)	ANOVA ME Group	ANOVA ME Time	ANOVA ME Drink	ANOVA Group x Time	ANOVA Group x Drink	ANOVA Time x Drink	ANOVA Group x Time x Drink
PHE BAL PRE	53.69 ± 6.63	53.17 ± 5.31							
PHE BAL POST	162.62 ± 55.44	128.75 ± 50.45							
PHE APTD PRE	51.69 ± 5.19	53.33 ± 5.65							
PHE APTD POST	11.15 ± 5.44	10.17 ± 4.02	F(1)=2.29, p=0.14, η^2^=0.09	**F(1)=162.80, p<0.01, η^2^=0.88	**F(1)=23.57, p<0.01, η^2^=0.51	F(1)=2.71, p=0.11, η^2^=0.11	F(1)=3.00, p=0.10, η^2^=0.12	**F(1)=149.0, p<0.01, η^2^=0.87	F(1)=1.95, p=0.18, η^2^=0.08
TYR BAL PRE	49.39 ± 10.79	47.25 ± 7.03							
TYR BAL POST	127.08 ± 26.12	117.25 ± 38.03							
TYR APTD PRE	48.69 ± 7.98	50.83 ± 9.50							
TYR APTD POST	13.08 ± 3.09	13.42 ± 3.58	F(1)=0.29, p=0.59, η^2^=0.01	**F(1)=283.79, p<0.01, η^2^=0.93	**F(1)=50.78, p<0.01, η^2^=0.69	F(1)=1.28, p=0.27, η^2^=0.05	F(1)=0.82, p=0.37, η^2^=0.03	**F(1)=242.03, p < 0.01, η^2^=0.91	F(1)=0.17, p=0.68, η^2^=0.17

*Legend*: Plasma PHE and TYR concentrations observed at baseline (PRE) and 4 hours post amino acid drink consumption (POST). Results are reported for both the balanced (BAL) and the acute phenylalanine/tyrosine depletion (APTD) conditions. Data are expressed as Means ± SD. APTD resulted in a significant lowering of plasma PHE and TYR concentrations.

** *P < 0*.*01*.

AN REC: anorexia nervosa recovered. ANOVA: analysis of variance. HC: healthy controls. ME: main effect. SD: standard deviation. X: interaction effect.

### PR Exercise Breakpoint Task

**Fig 1:**
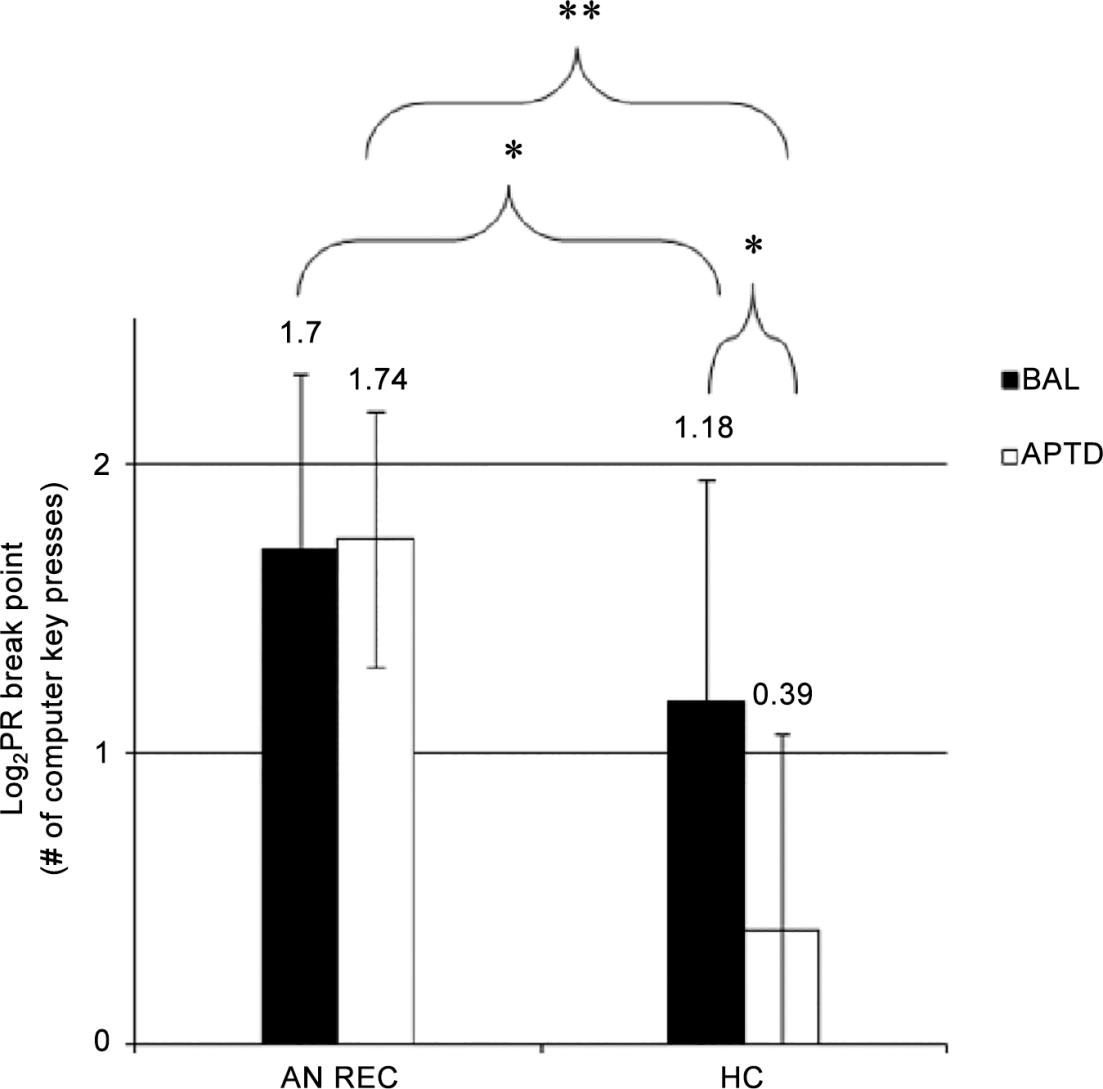
Effect of Acute Phenylalanine and Tyrosine Depletion (APTD) on Non-Transformed (A) and Log Transformed (B) Progressive Ratio (PR) Exercise Breakpoint Scores in Individuals Recovered from Anorexia Nervosa (AN REC, N = 17) and Healthy Controls (HC, N = 15). Relative to the balanced condition (BAL), APTD resulted in a significant decrease in PR breakpoint scores among HC only (HC BAL: 1.18 ± 0.76; HC APTD: 0.39 ± 0.68). Relative to HC, AN REC performed significantly more on the PR task during both BAL (1.70 ± 0.61) and APTD (1.74 ± 0.44). In AN REC, raw PR exercise breakpoint scores for were 113.53 ± 200.56 during BAL and 107.94 ± 200.76 during APTD. In HC, they were 36.87 ± 26.38 during BAL and 8.00 ± 14.49 during APTD. Data are expressed as Means ± SD. **P ≤ 0.*01* *P ≤ 0.*05*. ANOVA: analysis of variance. SD: standard deviation.

Task data were transformed logarithmically to satisfy the assumption of normality. Repeated measures ANOVA showed a significant Group by Drink interaction for PR exercise breakpoint scores [F(1, 30) = 15.70, p < 0.01, *η^2^* = 0.34] (Fig 1). Bonferroni corrected and bootstrap methods for *post-hoc* t-tests indicated that AN REC were more willing than HC to work for exercise in both the BAL [t (30) = -2.11, p = 0.05, ES = 0.77] and APTD conditions [t (30) = -6.58, p < 0.01, ES = 2.41]. Moreover, relative to BAL, APTD led to a significant decrease in breakpoint scores among HC [t (14) = 4.03, p < 0.01, ES = 1.47], an effect that was not produced in the AN REC [t (16) = -0.41, p = 0.70, ES = 0.26].

In AN REC, correlational analyses revealed a positive relationship between exercise breakpoint difference scores (*i*.*e*., PR breakpoint score during BAL–PR breakpoint score during APTD) and VAS “urge to exercise” ratings reported during BAL (r = 0.48, p = 0.05), suggesting that a higher drive to exercise is associated with increased willingness to work for exercise on a PR task, but importantly, only in the BAL condition. This relationship was not observed with VAS “urge to exercise” ratings during APTD (r = 0.40, p = 0.12) despite lower (albeit non-significant) self-reported urge to exercise ratings in this condition (1.65 ± 1.84) relative to BAL (2.49 ± 2.08). Weekly exercise frequency (hours/week) was positively correlated was PR exercise breakpoint difference scores in HC only (r = 0.60, p = 0.02). This suggests that sensitivity to APTD on willingness to work for physical activity was associated with weekly regular exercise in HC; in AN REC, however, willingness to work for exercise in both AA states occurred irrespective of current baseline exercise behaviour. Finally, REI-health scores were positively correlated with PR breakpoint difference scores in HC (r = 0.53, p = 0.05), suggesting that sensitivity to APTD on the PR task was associated with exercising to improve health in this group (S2 Table).

### APTD and Self-Reported Mood

Repeated measures ANOVAs showed main effects of Group for all DASS-21 subscores. For DASS–D scores, there was also a trend towards a main effect of Time. *Post-hoc* analyses revealed that AN REC tended to be more depressed (p = 0.07), anxious (p = 0.04), and stressed (p < 0.01) than HC. Moreover, DASS-D scores tended to increase over time irrespective of drink condition (p = 0.05) (Table 3). For results on the effects of APTD and Time on POMS-BI and VAS scores see S3 Table.

**Table 3:** Mood Over Time: DASS-21 Scores.

Mood	AN REC (n = 17)	HC (n = 15)	ANOVA ME Group	ANOVA ME Time	ANOVA ME Drink	ANOVA Group x Time	ANOVA Group x Drink	ANOVA Time x Drink	ANOVA Group x Time x Drink
DASS-D BAL PRE	6.59 ± 7.96	1.60 ± 2.41							
DASS-D BAL POST	7.29 ± 8.69	1.87 ± 2.20							
DASS-D APTD PRE	5.29 ± 5.15	2.67 ± 4.45							
DASS-D APTD POST	6.59 ± 7.74	4.27 ± 6.36	*F(1)=4.02, p=0.05, η^2^=0.12	F(1)=3.90, p=0.06, η^2^=0.12	F(1)=0.18, p=0.68, η^2^=0.01	F(1)=0.01, p=0.95, η^2^<0.01	F(1)=2.46, p=0.13, η^2^=0.08	F(1)=0.68, p=0.42, η^2^=0.02	F(1)=0.10, p=0.75, η^2^<0.01
DASS-A BAL PRE	5.88± 6.56	0.80 ±1.66							
DASS-A BAL POST	4.25 ± 5.74	1.20 ±1.66							
DASS-A APTD PRE	4.00 ± 4.12	1.07 ±1.98							
DASS-A APTD POST	5.18 ± 6.64	3.33 ±7.24	*F(1)=5.61, p=0.03, η^2^=0.16	F(1)=2.43, p=0.13, η^2^=0.08	F(1)=0.02, p=0.88, η^2^<0.01	F(1)=0.99, p=0.33, η^2^=0.03	F(1)=2.96, p=0.10, η^2^=0.09	F(1)=2.36, p=0.15, η^2^=0.07	F(1)<0.01, p=0.97, η^2^<0.01
DASS-S BAL PRE	15.06 ± 8.98	7.20 ±7.08							
DASS-S BAL POST	16.12 ± 7.53	6.27 ±6.76							
DASS-S APTD PRE	16.47 ± 10.04	6.40 ±7.02							
DASS-S APTD POST	16.12 ± 10.31	7.07 ±8.35	**F(1)=11.69, p<0.01, η^2^=0.28	F(1)=0.02, p=0.88, η^2^<0.01	F(1)=0.16, p=0.70, η^2^=0.01	F(1)=0.12, p=0.73, η^2^<0.01	F(1)=0.16, p=0.70, η^2^=0.01	F(1)=0.01, p=0.94, η^2^<0.01	F(1)=1.57, p=0.22, η^2^=0.05
DASS-T BAL PRE	27.53 ± 18.21	9.60 ± 9.89							
DASS-T BAL POST	28.71 ± 15.57	9.33 ± 9.16							
DASS-T APTD PRE	25.76 ± 16.40	10.13 ± 11.96							
DASS-T APTD POST	27.88 ± 21.33	14.67 ± 21.62	**F(1)=10.76, p<0.01, η^2^=0.28	F(1)=1.63, p=0.21, η^2^=0.05	F(1)=0.22, p=0.65, η^2^=0.01	F(1)=0.03, p=0.87, η^2^<0.01	F(1)=1.44, p=0.24, η^2^=0.05	F(1)=0.79, p=0.38, η^2^=0.03	F(1)=0.36, p=0.56, η^2^=0.01

*Legend*: The DASS-21 was administered at baseline (PRE) and following testing (POST) in both the balanced (BAL) and the acute phenylalanine/tyrosine depletion (APTD) conditions. Data are expressed as Means ± SD.

**P ≤ 0.*01*

*P ≤ 0.*05*.

AN REC: anorexia nervosa recovered. ANOVA: analysis of variance. DASS: Depression, Anxiety, and Stress Scales. DASS-D: DASS-Depression. DASS-A: DASS-Anxiety. DASS-S: DASS-Stress. DASS-T: DASS-Total. HC: healthy controls. ME: main effect. SD: standard deviation. X: interaction effect.

## Discussion

### APTD and Motivation to Obtain Exercise Reward

We investigated motivation to exercise in women recovered from AN and HCs under APTD and control conditions. Both groups worked for the opportunity to exercise, suggesting that physical activity can have greater reinforcing value than inactivity under the conditions of the current study; *e*.*g*., due to boredom or wanting to fulfil research expectancies. More importantly, the results also indicated that AN REC participants worked for higher PR breakpoints than HC, supporting suggestions that exercise has increased reinforcing efficacy in people with a history of AN [29, 30, 33]. The lowered DA state did not decrease willingness to work for exercise in AN REC but did in HCs. Breakpoint difference scores showed a positive relationship with self-reported urge to exercise in AN REC during the balanced condition only, suggesting that willingness to work for exercise during APTD occurred irrespective of decreased subjective impetus to exercise in this state. Similarly, sensitivity to APTD on willingness to work for exercise was associated with baseline exercise levels (hours/week) in HC only, suggesting that heightened drive to exercise in AN REC occurred irrespective of current weekly exercise patterns.

The effect of lowering DA on exercise breakpoints in HC is in accord with studies showing that APTD decreases willingness to work for drug and monetary rewards in healthy individuals [31, 36] and those with mild to moderate tobacco use disorders [32]. These effects likely occurred via decreases in reward-associated striatal DA release, an effect proposed to diminish the ability to sustain motivation to obtain reward [49]. This is consistent with the view that elevated DA transmission increases the ability of reward-related events to heighten motivational drive [50, 51] and suggests that voluntary exercise may, to some extent, be a DA-dependent natural reinforcer. In comparison, APTD did not decrease exercise breakpoints in AN REC, suggesting that pursuit of exercise was not as closely associated with DA transmission in these participants. Neuroimaging studies in individuals recovered from AN also suggest their responses to motivationally relevant stimuli are altered [8, 9, 12]. For example, compared to HC, AN REC women exhibit reduced differentiation of responses to rewards and punishments within the ventral striatum, and elevated reward-induced activations in bilateral caudate, dorsal-striatal, and cortical regions that project to these areas, suggesting a more strategic approach to reward-based tasks [9].

The present study suggests that in HC, drive to exercise may be a more purposeful, goal-directed act influenced by limbic DA transmission whereas in AN REC participants, it may reflect compulsive features associated with previous illness and regulated by substantially different neurocircuitry. For example, other compulsive behaviours seen in obsessive-compulsive disorder and addictions have been proposed to reflect heightened cortico-striatal excitatory input [17, 19, 42]. In such conditions, lowering DA within ventral circuitry may have less influence on maintaining ongoing behaviours. Consistent with this are preliminary data suggesting that DA depletion reduces preference for activity in mice with low but not high experiences of exercise [28] and that APTD shifts the balance from stimulus-reward goal-directed actions to stimulus-response habits in healthy women [38]. Thus, in AN REC, a lack of effect of APTD on drive to exercise may reflect the presence of automatic, habitual cognitive biases towards ED-related behaviours that have arisen from disorder-related reward associations [4, 17, 52]. Indeed, correlations between breakpoint difference scores and weekly exercise levels support this interpretation in that sensitivity to APTD on drive for exercise was moderated by weekly exercise patterns (*i*.*e*., regular exercise) in HC only, suggesting that individuals recovered from AN worked harder for exercise in both conditions irrespective of having a regular exercise regime or not.

Importantly however, dopaminergic reward systems are complex, being implicated in both the development of reward-based, goal-directed actions as well as compulsive, habit-like behaviours. Indeed, recent review of the evidence suggests that altered reward-based behaviour is reflected in the compulsive symptomatology characterising disorders such as AN, obsessive compulsive disorder, and substance dependence, with substantial overlap in the neural circuits underpinning these processes [17]. This may help explain why administration of DA receptor agonists in some patients is associated with the development of compulsive behaviours, including compulsive eating [53, 54]. Nevertheless, a more refined understanding of the transition from voluntary, reward-based behaviour to compulsive-like habit formation is, in part, neurally underpinned by a progression from ventral to dorsal striatal control, and thus expression of compulsive behaviour may be less dependent on alterations in dopaminergic reward circuitry innervating more ventral areas of the striatum [16–19, 55].

It is reasonable to hypothesise that AN REC are resistant to the effects of APTD because exercise has acquired elements of a compulsive habit not under motivational control. Alternative explanations are however plausible and may have accounted for the variance observed in PR exercise breakpoints scores. For example, enhanced achievement orientation (common in AN) might have buffered against effects of APTD: however, no group differences were observed in baseline levels of BAS-Drive and TCI-Persistence, both of which reflect a tendency to persevere in rewarding behaviours and goal-achievement. AN REC were more depressed, anxious, and stressed than HC and therefore these individuals may, to a different extent, use exercise as a means of improving mood. Similarly, reports of increased hunger prior to exercise as well as greater (non-significant) increases across time in non-clinical levels of depression/anxiety in HC make it possible that this group was less driven to work for exercise due to experiencing aversive subjective states. In addition, while data suggest that lowered DA may shift behaviour in favour of habits vs goals [38], this effect is likely dependent on the starting point of the system, which would dictate whether individuals with AN are more or less sensitive to DA manipulations. Thus, while our results indicate there are alterations in dopaminergic systems in AN, lack of information on baseline function in this system precludes definitive conclusions on the direction of such alterations. Finally, the data give rise to another important issue, namely that the AN REC showed comparable exercise hours/week as HC, and thus it remains difficult to explain how they might control their putative “habit” to exercise in daily life. This raises the issue of what other neural changes may need to occur from the acute stages of illness through to recovery. Changes in cognitive control processing are an obvious candidate but a more robust explanation will require comparative studies between ill and recovered individuals, both behaviourally and also involving neuroimaging in the ill and healthy states.

### Strengths and Limitations

This is the first study of the effects of APTD on motivation to exercise in AN. It involved AN REC participants, avoiding potentially confounding influences of malnutrition and weight-loss. The sample size was small but sufficient to obtain significant findings. The study has a few limitations. Only women were studied, and thus findings cannot be extrapolated to males. Women recovered from AN who were on a stable dose of SSRI medication were also included, potentially impacting on dopaminergic effects. Although no differences in exercise breakpoint scores were observed between AN REC who were taking antidepressants and those who were not, the sample size was small and thus it may not have been possible to detect whether antidepressants confounded the results.

Methodological differences make it difficult to compare data with other PR exercise tasks. A different PR schedule was used previously to measure the reinforcing efficacy of exercise in AN [33] and a different operant design may have yielded different findings. The PR task was not set-up to demonstrate an impact on any other rewarding behaviour; *i*.*e*., our conclusions could have been strengthened by an investigation into the effects of APTD on willingness to earn rewards not related to AN (e.g., money). Moreover, little information was available on which recovered participants showed hyperactivity while acutely ill, which could have impacted results on the exercise task. Similarly, given that groups were not matched for current regular exercise, reasons for exercise, or the degree of commitment to exercise, it is not possible to definitely conclude that findings relate specifically to AN or may also apply to individuals who engage in habitual exercise routines. Inclusion of healthy athlete controls would therefore be of added value. Moreover, PET and neuroendocrine studies suggest that APTD decreases DA release by 30 to 50% [56, 57]. Larger effects are seen under challenge conditions [58], and, in microdialysis studies conducted in rats, stimulated DA release can be decreased by up to 70% [59]. Some behavioural effects, though, might require larger DA decrements. Finally, APTD might affect the metabolism of catecholamines other than DA (i.e., norepinephrine) [60]; however, microdialysis [59, 61], neuroendocrine [56, 62, 63], and fos immunocytochemical studies [64] indicate that it preferentially affects DA transmission.

## Conclusions

This study supports the feasibility and utility of using a PR exercise breakpoint task to assess the reinforcing value of exercise in ED. The data provide evidence that lowered DA transmission reduces the motivational value of activity in HC but not in AN REC. Thus in AN, elevated drive to exercise may occur independent of dopaminergic reward processes. Egosyntonic beliefs associated with AN are a major barrier to treatment and recovery; therefore, further studies on the mechanisms and stimuli underpinning the compulsive pursuit of illness-related rewards are needed.

## Supporting Information

S1 Table Participant Baseline Characteristics. Data are expressed as Means ± SD. ** *P < 0*.*01*. * P < 0.*05*. AN REC: anorexia nervosa recovered. BIS/BAS: The Behavioural Inhibition (BIS) and Behavioural Activation (BAS) Scales. ES: Cohen’s d effect size. HC: healthy controls. SPSRQ: The Sensitivity to Punishment and Sensitivity to Reward Questionnaire. TCI-R: The Temperament and Character Inventory Revised. SD: standard deviation.
(DOCX)

S2 Table Pearson Correlation Analyses for the Progressive Ratio (PR) Exercise Breakpoint Difference Scores With Baseline Characteristics. Correlations between log transformed PR exercise breakpoint difference scores (PR breakpoint score during the balanced (BAL) condition–PR breakpoint score during the acute phenylalanine/tyrosine depletion (APTD) condition) and self-report measures of eating pathology, mood, baseline exercise experience, reasons for exercise, achievement orientation (e.g., drive and persistence), reward and punishment sensitivity, hunger, and nausea. **P ≤ 0.*01* *P ≤ 0.*05*. BIS/BAS: The Behavioural Inhibition (BIS) and Behavioural Activation (BAS) Scales. BMI: Body Mass Index. DASS: Depression, Anxiety, and Stress Scales. EDE-Q: Eating Disorders Examination Questionnaire. POMS: Bipolar Profile of Mood States. REI: Reasons for Exercise Inventory. TCI-R: Temperament and Character Inventory, Revised. VAS: Visual Analogue Scales.
(DOCX)

S3 Table Mood Over Time: POMS-BI Scores. The POMS-BI was administered at baseline (PRE) and following testing (POST). Data are expressed as Means ± SD. **P ≤ 0.*01* *P ≤ 0.*05*. AN REC: anorexia nervosa recovered. ANOVA: analysis of variance. HC: healthy controls. ME: main effect. POMS-BI: Bipolar Profile of Mood States. SD: standard deviation. X: interaction effect.
(DOCX)
