# Supplementary material for: The Effects of Acute Dopamine Precursor Depletion on the Reinforcing Value of Exercise in Anorexia Nervosa
Source: PLoS One. 2016 Jan 25;11(1):e0145894. doi: 10.1371/journal.pone.0145894 (PMC4726788; doi:10.1371/journal.pone.0145894)
Supplement: S1 Table — Data are expressed as Means ± SD. ** P < 0.01. * P < 0.05. AN REC: anorexia nervosa recovered. BIS/BAS: The Behavioural Inhibition (BIS) and Behavioural Activation (BAS) Scales. ES: Cohen’s d effect size. HC: healthy controls. SPSRQ: The Sensitivity to Punishment and Sensitivity to Reward Questionnaire. TCI-R: The Temperament and Character Inventory Revised. SD: standard deviation. (DOCX) [file pone.0145894.s001.docx]

## S1 Table. Participant baseline characteristics

| **Baseline Characteristics** | **AN REC (n = 17)** | **HC (n = 15)** | **Statistics: AN REC to HC** |
| --- | --- | --- | --- |
| TCI-R, Novelty Seeking | 95.08 ± 23.67 | 107.86 ± 8.85 | t (25) = 1.83, p = 0.09, ES = 0.67 |
| TCI-R, Harm Avoidance * | 111.77 ± 28.21 | 93.71 ± 12.16 | t (25) = -2.13, p = 0.05, ES = 0.78 |
| TCI-R, Reward Dependence | 107.64 ± 12.83 | 111.77 ± 28.21 | t (25) = 1.62, p = 0.12, ES = 0.59 |
| TCI-R, Persistence | 139.46 ± 17.53 | 128.71 ± 11.34 | t (25) = -1.91, p = 0.07, ES = 0.70 |
| TCI-R, Self-Directedness | 136.15 ± 21.38 | 147.57 ± 14.21 | t (25) = 1.65, p = 0.11, ES = 0.60 |
| TCI-R, Cooperativeness | 138.77 ± 9.87 | 139.64 ± 12.29 | t (25) = 0.20, p = 0.84, ES = 0.07 |
| TCI-R, Self-Transcendence | 54.31 ± 16.61 | 64.29 ± 13.85 | t (25) = 1.70, p = 0.10, ES = 0.62 |
| BAS, Drive | 10.97 ± 2.59 | 10.93 ± 1.91 | t (30) = -0.05, p = 0.96, ES = 0.02 |
| BAS, Fun Seeking | 10.59 ± 2.22 | 11.70 ± 2.08 | t (30) = 1.46, p = 0.16, ES = 0.53 |
| BAS, Reward Responsiveness | 16.44 ± 2.23 | 16.27 ± 3.13 | t (30) = -0.18, p = 0.86, ES = 0.07 |
| BAS, Total Score | 38.03 ± 5.53 | 38.90 ± 6.12 | t (30) = 0.42, p = 0.68, ES = 0.15 |
| BIS, Anxiety | 16.65 ± 2.94 | 14.63 ± 3.48 | t (30) = -1.78, p = 0.09, ES = 0.65 |
| BIS, Fear * | 5.97 ± 0.98 | 5.23 ± 1.03 | t (30) = -2.08, p = 0.05, ES = 0.76 |
| BIS, Total Score | 22.62 ± 3.58 | 19.87 ± 4.28 | t (30) = -1.98, p = 0.06, ES = 0.72 |

*Legend:* Data are expressed as Means ± SD. *** P < 0.01. * P < 0.05.* AN REC: anorexia nervosa recovered. BIS/BAS: The Behavioural Inhibition (BIS) and Behavioural Activation (BAS) Scales. ES: Cohen’s d effect size. HC: healthy controls. SPSRQ: The Sensitivity to Punishment and Sensitivity to Reward Questionnaire. TCI-R: The Temperament and Character Inventory Revised. SD: standard deviation.
