# Supplementary material for: The Effects of Acute Dopamine Precursor Depletion on the Reinforcing Value of Exercise in Anorexia Nervosa
Source: PLoS One. 2016 Jan 25;11(1):e0145894. doi: 10.1371/journal.pone.0145894 (PMC4726788; doi:10.1371/journal.pone.0145894)
Supplement: S2 Table — Correlations between log transformed PR exercise breakpoint difference scores (PR breakpoint score during the balanced (BAL) condition–PR breakpoint score during the acute phenylalanine/tyrosine depletion (APTD) condition) and self-report measures of eating pathology, mood, baseline exercise experience, reasons for exercise, achievement orientation (e.g., drive and persistence), reward and punishment sensitivity, hunger, and nausea. **P ≤ 0.01 *P ≤ 0.05. BIS/BAS: The Behavioural Inhibition (BIS) and Behavioural Activation (BAS) Scales. BMI: Body Mass Index. DASS: Depression, Anxiety, and Stress Scales. EDE-Q: Eating Disorders Examination Questionnaire. POMS: Bipolar Profile of Mood States. REI: Reasons for Exercise Inventory. TCI-R: Temperament and Character Inventory, Revised. VAS: Visual Analogue Scales. (DOCX) [file pone.0145894.s002.docx]

## S2 Table. Pearson correlation analyses for the progressive ratio (PR) exercise breakpoint difference scores with baseline characteristics

| **Baseline Characteristics** | **AN REC (n = 17)** | **HC (n = 15)** | **Correlation with Log PR breakpoint difference score for AN REC** | **Correlation with Log PR breakpoint difference score for HC** |
| --- | --- | --- | --- | --- |
| BMI | 21.45 ± 2.13 | 21.74 ± 1.58 | r = 0.05, p = 0.84 | r = 0.47, p = 0.08 |
| EDE-Q, Global | 1.55 ± 1.01 | 0.38 ± 0.34 | r = -0.12, p = 0.65 | r = -0.15, p = 0.60 |
| DASS, Total | 26.65 ± 16.43 | 9.87 ± 10.18 | r = -0.32, p = 0.22 | r = -0.32, p = 0.25 |
| Exercise Frequency, hours/week | 3.69 ± 2.52 | 2.64 ± 1.46 | r = -0.08, p = 0.78 | r = 0.60*, p = 0.02 |
| REI, Weight Control | 4.59 ± 1.50 | 3.62 ± 1.49 | r = -0.34, p = 0.18 | r = 0.36, p = 0.20 |
| REI, Attractiveness | 3.57 ± 1.42 | 3.88 ± 1.42 | r = -0.06, p = 0.81 | r = -0.13, p = 0.66 |
| REI, Tone | 4.55 ± 1.58 | 2.73 ± 1.57 | r = -0.03, p = 0.92 | r = 0.14, p = 0.63 |
| REI, Health | 4.57 ± 1.18 | 5.16 ± 1.50 | r = 0.42, p = 0.09 | r = 0.53*, p = 0.05 |
| REI, Fitness | 5.13 ± 1.13 | 4.93 ± 1.18 | r = 0.22, p = 0.40 | r = 0.17, p = 0.57 |
| REI, Mood | 4.60 ± 1.21 | 4.00 ± 1.65 | r = -0.09, p = 0.73 | r = 0.13, p = 0.65 |
| REI, Enjoyment | 2.49 ± 1.04 | 2.76 ± 1.80 | r = 0.07, p = 0.79 | r = 0.33, p = 0.24 |
| TCI-R, Novelty Seeking | 95.08 ± 23.67 | 107.86 ± 8.85 | r = 0.49, p = 0.09 | r = -0.28, p = 0.34 |
| TCI-R, Harm Avoidance | 111.77 ± 28.21 | 93.71 ± 12.16 | r = -0.29, p = 0.33 | r = -0.25, p = 0.38 |
| TCI-R, Reward Dependence | 99.85 ± 12.07 | 107.64 ± 8.85 | r = 0.20, p = 0.51 | r = 0.46, p = 0.10 |
| TCI-R, Persistence | 139.46 ± 17.53 | 128.71 ± 11.34 | r = -0.03, p = 0.92 | r = 0.04, p = 0.90 |
| TCI-R, Self-Determination | 136.15 ± 21.38 | 147.57 ± 12.21 | r = -0.02, p = 0.96 | r = 0.24, p = 0.41 |
| BAS, Drive | 10.97 ± 2.59 | 10. 93 ± 1.91 | r = 0.09, p = 0.73 | r = -0.42, p = 0.12 |
| BAS, Fun Seeking | 10.59 ± 2.22 | 11.70 ± 2.08 | r = -0.12, p = 0.65 | r = 0.06, p = 0.83 |
| BAS, Reward Responsiveness | 16.44 ± 2.23 | 16.27 ± 3.13 | r = 0.29, p = 0.26 | r = -0.16, p = 0.57 |
| BAS, Total Score | 38.03 ± 5.53 | 38.90 ± 6.12 | r = 0.11, p = 0.67 | r = -0.19, p = 0.50 |
| BIS, Anxiety | 16.65 ± 2.94 | 14.63 ± 3.48 | r = 0.30, p = 0.24 | r = -0.32, p = 0.24 |
| BIS, Fear | 5.97 ± 0.98 | 5.23 ± 1.03 | r = 0.18, p = 0.50 | r = -0.19, p = 0.50 |
| BIS, Total Score | 22.62 ± 3.58 | 19.87 ± 4.28 | r = 0.30, p = 0.25 | r = -0.31, p = 0.26 |
| VAS Urge to Exercise BAL | 2.49 ± 2.08 | 1.64 ± 2.32 | r = 0.48*, p = 0.05 | r = 0.14, p = 0.61 |
| VAS Urge to Exercise APTD | 1.65 ± 1.84 | 1.21 ± 2.26 | r = 0.42, p = 0.10 | r = 0.06, p = 0.85 |
| VAS Hunger BAL | 5.57 ± 1.97 | 7.12 ± 2.92 | r = -0.03, p = 0.92 | r = 0.22, p = 0.43 |
| VAS Hunger APTD | 6.12 ± 2.69 | 6.65 ± 2.71 | r = -0.01, p = 0.99 | r = -0.23, p = 0.43 |
| VAS Nausea BAL | 2.41 ± 2.71 | 2.24 ± 2.58 | r = -0.29, p = 0.26 | r = -0.29, p = 0.30 |
| VAS Nausea APTD | 4.44 ± 2.76 | 4.66 ± 3.64 | r = -0.08, p = 0.76 | r = -0.04, p = 0.90 |

*Legend:* Correlations between log transformed PR exercise breakpoint difference scores (PR breakpoint score during the balanced (BAL) condition – PR breakpoint score during the acute phenylalanine/tyrosine depletion (APTD) condition) and self-report measures of eating pathology, mood, baseline exercise experience, reasons for exercise, achievement orientation (e.g., drive and persistence), reward and punishment sensitivity, hunger, and nausea. ***P ≤ 0.01* **P ≤ 0.05.* BIS/BAS: The Behavioural Inhibition (BIS) and Behavioural Activation (BAS) Scales. BMI: Body Mass Index. DASS: Depression, Anxiety, and Stress Scales. EDE-Q: Eating Disorders Examination Questionnaire. POMS: Bipolar Profile of Mood States. REI: Reasons for Exercise Inventory. TCI-R: Temperament and Character Inventory, Revised. VAS: Visual Analogue Scales.
