# Supplementary material for: The Effects of Acute Dopamine Precursor Depletion on the Reinforcing Value of Exercise in Anorexia Nervosa
Source: PLoS One. 2016 Jan 25;11(1):e0145894. doi: 10.1371/journal.pone.0145894 (PMC4726788; doi:10.1371/journal.pone.0145894)
Supplement: S3 Table — The POMS-BI was administered at baseline (PRE) and following testing (POST). Data are expressed as Means ± SD. **P ≤ 0.01 *P ≤ 0.05. AN REC: anorexia nervosa recovered. ANOVA: analysis of variance. HC: healthy controls. ME: main effect. POMS-BI: Bipolar Profile of Mood States. SD: standard deviation. X: interaction effect. (DOCX) [file pone.0145894.s003.docx]

## S3 Table. Mood over time: POMS-BI scores

| **POMS subscale** | | **AN REC (n = 17)** | **HC (n = 15)** | **ANOVA ME Group** | | **ANOVA ME Time** | **ANOVA ME Drink** | **ANOVA Group x Time** | **ANOVA Group x Drink** | **ANOVA Time x Drink** | **ANOVA Group x Time x Drink** |
| --- | --- | --- | --- | --- | --- | --- | --- | --- | --- | --- | --- |
| Composed/Anxious BAL | PRE | 21.25 ± 6.64 | 29.54 ± 5.90 | * F(1) = 5.84 p = 0.02 η^2^ = 0.18 | | ** F(1) = 10.24 p < 0.01 η^2^ = 0.28 | F(1) = 2.41 p = 0.13 η^2^ = 0.08 | * F(1) = 6.73 p = 0.02 η^2^ = 0.20 | * F(1) = 4.04 p = 0.05 η^2^ = 0.13 | ** F(1) = 8.39 p = 0.01 η^2^ = 0.24 | F(1) = 2.00 p = 0.17 η^2^ = 0.07 |
|  | POST | 21.75 ± 7.72 | 27.46 ± 6.55 |  |  |  |  |  |  |  |  |
| Composed/Anxious APTD | PRE | 22.69 ± 5.87 | 29.38 ± 5.77 |  |  |  |  |  |  |  |  |
|  | POST | 21.13 ± 8.35 | 21.31 ± 7.87 |  |  |  |  |  |  |  |  |
| Elated/ Depressed BAL | PRE | 18.19 ± 5.76 | 23.77 ± 5.83 | * F(1) = 6.11 p = 0.02 η^2^ = 0.19 | | F(1) = 1.04 p = 0.32 η^2^ = 0.04 | F(1) = 0.01 p = 0.93 η^2^ < 0.01 | F(1) = 1.20 p = 0.28 η^2^ = 0.04 | F(1) = 1.03 p = 0.32 η^2^ = 0.04 | ** F(1) = 10.58 p < 0.01 η^2^ = 0.28 | F(1) = 1.73 p = 0.20 η^2^ = 0.06 |
|  | POST | 19.38 ± 6.26 | 24.77 ± 4.64 |  |  |  |  |  |  |  |  |
| Elated/ Depressed APTD | PRE | 20.06± 5.69 | 25.54 ± 4.35 |  |  |  |  |  |  |  |  |
|  | POST | 19.00 ± 8.33 | 21.23 ± 6.46 |  |  |  |  |  |  |  |  |
| Energetic/Tired  BAL | PRE | 13.44 ± 8.99 | 16.15 ± 8.47 | F(1) = 0.37 p = 0.55 η^2^ = 0.01 | | * F(1) = 5.07 p = 0.03 η^2^ = 0.16 | F(1) = 0.53 p = 0.47 η^2^ = 0.02 | F(1) = 1.99 p = 0.17 η^2^ = 0.07 | F(1) = 0.02 p = 0.89 η^2^ < 0.01 | ** F(1) = 23.07 p < 0.01 η^2^ = 0.46 | F(1) = 0.47 p = 0.50 η^2^ = 0.02 |
|  | POST | 15.19 ± 9.53 | 15.92 ± 7.68 |  |  |  |  |  |  |  |  |
| Energetic/ Tired APTD | PRE | 15.19± 9.03 | 18.38 ± 8.72 |  |  |  |  |  |  |  |  |
|  | POST | 11.23 ± 7.09 | 11.23 ± 7.09 |  |  |  |  |  |  |  |  |
| Agreeable/ Hostile BAL | PRE | 25.69 ± 4.69 | 26.77 ± 5.88 | | F(1) = 0.46 p = 0.50 η^2^ = 0.02 | * F(1) = 5.70 p = 0.02 η^2^ = 0.16 | F(1) = 2.45  p = 0.13 η^2^ = 0.08 | F(1) = 0.79 p = 0.38 η^2^ = 0.03 | F(1) = 0.01 p = 0.93 η^2^ < 0.01 | * F(1) = 4.43 p = 0.04 η^2^ = 0.13 | F(1) = 0.52 p = 0.48 η^2^ = 0.02 |
|  | POST | 25.50 ± 4.26 | 26.38 ± 4.75 | |  |  |  |  |  |  |  |
| Agreeable/ Hostile APTD | PRE | 25.44 ± 2.80 | 27.92 ± 4.23 | |  |  |  |  |  |  |  |
|  | POST | 24.63 ± 4.87 | 24.69 ± 5.99 | |  |  |  |  |  |  |  |
| Confident/ Unsure BAL | PRE | 16.38 ± 8.07 | 21.38 ± 2.88 | | F(1) = 3.33 p = 0.08 η^2^ = 0.11 | F(1) = 3.64 p = 0.07 η^2^ = 0.12 | F(1) = 0.48 p = 0.50 η^2^ = 0.02 | F(1) = 2.31 p = 0.14 η^2^ = 0.08 | F(1) = 0.42 p = 0.52 η^2^ = 0.02 | ** F(1) = 13.88 p < 0.01 η^2^ = 0.01 | F(1) = 0.69 p = 0.42 η^2^ = 0.03 |
|  | POST | 17.63 ± 7.72 | 21.77 ± 4.05 | |  |  |  |  |  |  |  |
| Confident/ Unsure APTD | PRE | 17.81 ± 6.95 | 22.77 ± 4.92 | |  |  |  |  |  |  |  |
|  | POST | 16.13 ± 8.05 | 18.54 ± 6.16 | |  |  |  |  |  |  |  |
| Clear/ Confused BAL | PRE | 23.06 ± 7.22 | 26.23 ± 4.34 | | F(1) = 1.43 p = 0.24 η^2^ = 0.05 | ** F(1) = 17.62 p < 0.01 η^2^ = 0.40 | F(1) = 0.33 p = 0.57 η^2^ = 0.01 | F(1) = 1.34 p = 0.26 η^2^ = 0.05 | F(1) = 0.87 p = 0.36 η^2^ = 0.03 | ** F(1) = 8.98 p = 0.01 η^2^ = 0.25 | F(1) = 1.09 p = 0.30 η^2^ = 0.04 |
|  | POST | 22.13 ± 6.91 | 24.92 ± 4.70 | |  |  |  |  |  |  |  |
| Clear/ Confused APTD | PRE | 24.44 ± 5.19 | 27.31 ± 4.69 | |  |  |  |  |  |  |  |
|  | POST | 21.31 ± 5.99 | 21.46 ± 7.09 | |  |  |  |  |  |  |  |

*Legend:* The POMS-BI was administered at baseline (PRE) and following testing (POST). Data are expressed as Means ± SD. ***P ≤ 0.01* **P ≤ 0.05.* AN REC: anorexia nervosa recovered. ANOVA: analysis of variance. HC: healthy controls. ME: main effect. POMS-BI: Bipolar Profile of Mood States. SD: standard deviation. X: interaction effect.
